# Naso-Pharyngeal Catarrh

**Published:** 1880-09

**Authors:** 


### BIBLIOGRAPHICAL.

NASO-PHARYNGEAL CATARRH.—By Martin F. Comers, M.D., Professor of Ophthalomo'gy and Otology in the Kentucky School of Medicine, Etc., Etc. Bradley & Gilbert, Publishers, Louisville, Ky. 1880.

On account of the general unsatisfactory results in the treatment of this wide-spread and disagreeable disease, we are inclined to greet with avidity any suggestions or treatise on its treatment.

While in the majority of cases lack of success may be referable more to lack of promptness and perseverance in the patient, often the limited inability of the physician is the primary element of failure. We have seen patients treated persistently for a long time, and by the most approved methods extant, with negative result.

The partial inaccessability of the seat of the disease, with the peculiar nature of the tissue involved, renders the affection essentially difficult to cure.

In the work before us, in connection with a description of the general and minute anatomy of the parts affected, there is presented original and collected methods of treatment with individual apparatus and appliances. We doubt not the book will meet a hearty welcome, and may do much good.

